# Improving Biosecurity Procedures to Minimize the Risk of Spreading Pathogenic Infections Agents After Carcass Recycling

**DOI:** 10.3389/fmicb.2020.00623

**Published:** 2020-04-22

**Authors:** Pramod Pandey, Sriram K. Vidyarthi, Venkata Vaddella, Chandrasekar Venkitasamy, Maurice Pitesky, Bart Weimer, Alda F. A. Pires

**Affiliations:** ^1^Department of Population Health and Reproduction, University of California, Davis, Davis, CA, United States; ^2^Department of Biological and Agricultural Engineering, University of California, Davis, Davis, CA, United States; ^3^The Morning Star Company, Woodland, CA, United States

**Keywords:** infectious agents, BSE, *Salmonella*, *E. coli*, *Listeria*

## Abstract

Animal proteins are essential elements of human and animal feed chain and improving the safety of human and animal feed requires understanding and controlling of the transmission of infectious agents in food chain. Many pathogenic infectious agents, such as prion protein is known to damage the central nervous system in the cattle. Bovine spongiform encephalopathy (BSE) results from infection agent (prion), and affects number of species such as cattle, human, and cats. In addition, *Salmonella*, pathogenic *E. coli* O157:H7, and *Listeria* monocytogenes were found in animal by-products used in the human and animal feed production. Increased interest in controlling microbial risks in human and animal feed is evidenced by a large number of publications, which highlights the need for examining the animal disposal method such as rendering process and provides a broader perspective of rendering process. While existing practices help greatly in controlling microbial contamination, this overview study showed that additional biosafety measures are necessary to ensure microbial safety in animal feed.

## Introduction

Rendering industry is essential for disposing animal carcasses during routine and catastrophic mortality of animals. The disposal of animal carcasses during a large-scale mortality and outbreaks is an issue in the United States (US), and as well as in other countries. Data show that the total average annual losses of cattle, calves, and swine heads in the US are 8.7, 10.9, and 45.2 million, respectively (USDA-NASS, 2015). In addition, a total of 2.1 billion pounds of poultry including ante and post-mortem were lost between 2010 and 2014 (USDA-NASS, 2015). Controlling the potential risks to the public health and the human and animal feed chain requires the disposal of carcasses of dead animals within 36–40 h using special liquid tight transport vehicles/containers. Currently, many methods such as burial, composting, incineration, and rendering are the common practices for disposing dead animals during routine and catastrophic events (CAST, 2008a, b, 2009). Not all these disposal methods, however, are allowed in all states in the US due to the potential risks to human and animal feed chain and public health. For example, in California burial and composting of animal mortalities are generally the least opted methods, and are not allowed under certain circumstances. This highlights the importance of examining other disposal method such as rendering (CAL-EPA, 2004).x

Rendering of animal carcasses is used in several states in the US for managing routine animal mortality because it offers quick disposal with a reduced risk to environment (Hamilton and Meeker, 2006; Meeker and Hamilton, 2006; NRA, 2015). In routine livestock mortality management, day-to-day safe disposal of carcass is needed, which is relatively more predictable in terms of disposal capacity requirement compared to catastrophic outbreak events. Rendering is often used for managing routine mortality for preventing disease transmission and to protect water and air quality.

In contrast, catastrophic and outbreak events are often unpredictable and under those situations a sustainably larger numbers of animal carcasses requires safe disposal in limited time. The capacities of existing rendering facilities are built based on the need for routine carcass disposal are often dwarfed by production of carcasses during outbreak events. The limited capacity of rendering facilities to dispose animal carcasses during outbreak is a major challenge. The use of rendering facilities, which are built with the main objective of producing by products from animal carcasses, for tackling carcasses of animal during outbreaks faces multiple challenges in terms of by-product safety, cross contamination, and occurring of economic losses.

Further, the temperature and time, and processing method used in rendering process for producing by-products may not be sufficient to eradicate pathogens completely during outbreaks. In those situations, the combination of rendering with incineration is considered be a suitable disposal method (CAST, 2008b). In the past, the rendered products were incinerated to prevent the disease dissemination and contamination. The combination of rendering and incineration was used for dealing mortality in Asia and Europe. For example, during the 1997 foot and mouth disease (FMD) outbreak in Taiwan, 2001 FMD outbreak in the UK, and the 2001 FMD epidemic in the Netherlands, the rendered product was subsequently incinerated (CAST, 2008b).

In addition to the US, rendering is also common in European and Asian countries (Pandey et al., 2016). It is widely considered that rendering of animal carcasses during routine and catastrophic mortality is an environmentally safe method for disposing the dead livestock. One of the major advantages of rendering is that, it has a potential to destroy pathogens because of high temperature involved in rendering process (Pandey et al., 2016). Further, it produces usable end products such as meal, meat, melted fat or tallow, feather, bone, and blood meal which has potential to be used in animal feeds. However, the end products that are produced from keratin materials of carcasses such as hooves and horns are considered inedible for animals and are not allowed as animal feed. These inedible materials are often used as a fertilizer in many places. It is required that the prohibited by-products such as meat and bone meal must be labeled, recorded and controlled for use as a fertilizer. The by-product such as tallow is used for livestock feed, fatty acid production, and soap manufacturing (Sander et al., 2002; USDA-APHIS, 2004).

Livestock mortality is a great source of by-products that consist of protein and dry matter (DM). Rendered products are used as a source of organic matter for both animal feed and fertilizer (Pandey et al., 2016). In addition, it is used for grease production on an industrial level. Typically, a fresh carcass comprises about 32% DM which mainly contains ∼52% protein, 41% fat, and 6% ash. Rendering process offers the benefits of sourcing the organic matters, especially protein to the animal feed mill operations, and provides an option for recycling organic matter and protein. Product innovation and new food product development based on consumers’ demand is a key in food processing for sustainable growth (Vidyarthi et al., 2019a,b,c,d) and rendering process offers that benefits. Rendering process produces a range of products both edible and inedible animal proteins such as livestock feed, pet food and treats, soaps, pharmaceuticals, lubricants, plastics, soaps and shampoo, lotion, rubber, candles, candy, tallow, and lard (Pearl, 2005; Hamilton and Meeker, 2006; Swisher, 2014). The inedible rendering process converts the fat, protein, and keratin (i.e., hoof and horn) materials found in dead carcasses into inedible tallow, carcass meal, and fertilizers, respectively (Kalbasi-Ashtari et al., 2008).

**FIGURE 1:**
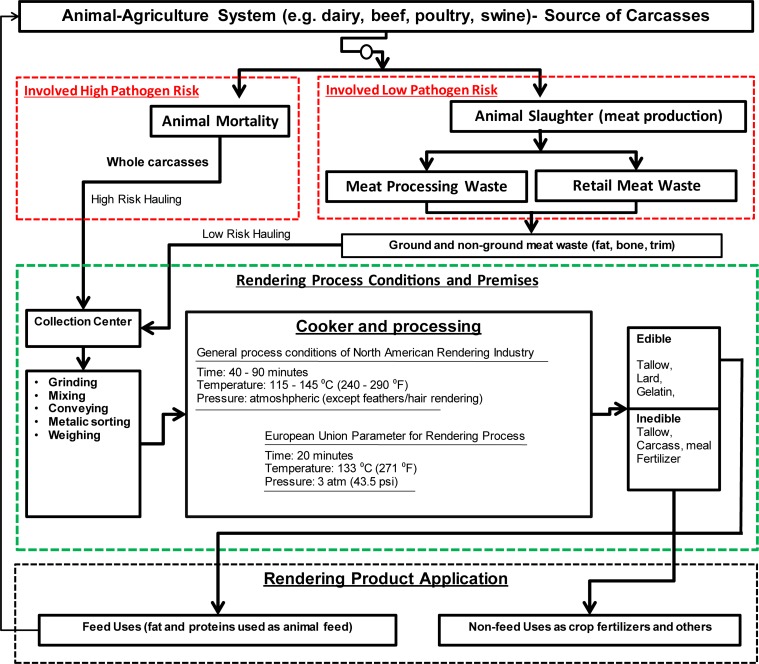
Conceptual flow of processes involved in rendering, and by-product productions (boxes under red dashed-line describe feedstock inflow, boxes under green dashed-line describe elements of rendering process, and boxes under black dashed-line indicates product application).

Rendered products are produced through a relatively complex rendering process. In rendering, multiple steps are involved for converting carcasses into useful by-products. The two major steps in rendering are: (1) mechanical defragmentation (i.e., grinding, mixing, pressing, decanting, and separating); and (2) heating (i.e., cooking, evaporating, and drying). Sometimes, chemical processes (solvent extraction) are also used (USDA-APHIS, 2004). During rendering, the carcasses are exposed to a pressurized steam often in the batch mode, which facilitates killing of pathogens. In general, heating to the temperatures of 115–145°C for 40–90 min are used for processing the feedstock in the US rendering industry (Pandey et al., 2016). In European Union (EU) Countries, pressurized (pressure ≈ 3 atm) cooking (temperature = 139°C) for 20 min is common (Figure 1).

In the process, a large-scale material inflows and by-product outflows are involved (Figure 1), and each step requires certain bio-safety measures to reduce the risk of dispersion of infectious agents. Depending on the targeted end products, the rendering process steps and relevant equipment may vary. Commonly used components in a rendering facility are particle size reduction equipment, separator, wedge-wire screens, centrifuges, steam generator, mechanical extractors, cookers, coolers, condensers, mechanical presses, grinders, dryers, filter, and deodorizers (Hamilton et al., 2012; US Environmental Protection Agency (US EPA), 2020). In terms of feedstocks, the two major sources of feedstock entering rendering facility are dead animals and slaughter house wastes produced in meat industry (Figure 1). In many situations, rendering of poultry carcasses are not conducted with mammals’ carcasses because the feathers require a higher temperature, and elevated temperatures during rendering increase the damages of proteins in mammals’ carcasses (Ritz, 2017). At high temperature, various waste streams including animal carcasses, slaughter house wastes such as bone, blood, fat trim, feathers, meat, viscera, and used cooking oils from food chain are processed in a rendering plant.

In general, the carcass rendering is considered environmentally safe because high temperature has a potential to eliminate pathogens. However, the transportation of livestock mortalities to the rendering plants poses biosecurity concerns and has a potential to spread animal diseases. Transportation of dead animals particularly during outbreaks may cause biosecurity risk because of large distances between rural areas, limited access to rendering facilities, and lack of timely pick up services of dead animals (Hammond, 1994; Crews et al., 1995; Bagley et al., 1999; Fonstad et al., 2003; Glanville et al., 2009).

It is important to consider the infectious agents, while selecting the disposal methods for dead animal carcasses. For example, if agent is BSE/scrapie, preferred disposal methods are burning, alkaline hydrolysis, and burying. These agents are classified as prion and non-viral. In case if agent is virus such as avian influence/Newcastle/foot and mouth disease/swine vesicular disease, the preferred disposal method is burying and burning. In vector born categories such as vesicular stomatitis, the preferred disposal method is burying and burning. If the agent is bacterial spore such as anthrax, burning is considered as preferred method (ARMCANZ, 2000; Geering et al., 2001; USDA-APHIS, 2004).

In general, rendering is a well accepted method for disposing carcasses. However, there is always a concern whether additional carcasses produced during catastrophic mortality can be absorbed in existing rendering facility (Missouri Department of Agriculture, 2008). In the U.S., a recent action report suggest to improve existing rendering processes to make them more suitable to tackle the carcasses during outbreaks (United States Department of Agriculture (USDA), 2017). It is has been suggested that rendering is a possible solutions for disposal of carcasses during a disease outbreaks. However, a number of issues such as biosecurity related to liability for potentially spreading disease, transportation of carcasses, returning a facility to full commercialization after processing infected animals, perceptions and communication, and compensation requires better understanding to engage existing rendering facilities for carcass disposal during outbreaks (United States Department of Agriculture (USDA), 2017).

In other parts of the world, for example, in Australia the law prohibits leaving carcasses to rot or dumping them in waterways. It is illegal to allow anyone other than a licenses knackery to remove meat from a farm. Disposal methods such as composting for dairy farms, burning in the case of emergency diseases, burial of livestock, and knackery are recommended to tackle cattle carcasses (Dairy Australia, 2016). Australian Veterinary Emergency Plan (AUSVETPLAN, 2008) outlines a series of methods such as burial, rendering, burning, composting for disposal and decontamination of carcasses during outbreaks and routine mortality. In the European Union, a series of criteria has been developed to categorize animal by products, and the disposal methods are suggested based on the animal by products categories. One main criteria is based on whether animal by-products will be used for human or animal consumptions or not. If by-products is used for human and animal, then the risks needs to be adequately controlled to reduce the risks of public and animal health. The disposal of all animal by-products is considered to be not a realistic option as it would involve unsustainable costs and risks to the environment (European Parliament, 2009).

## Rendered Products

The by-products of rendering process provide economic values (USDA-APHIS, 2004). Products such as tallow, lard, grease, fat, and meal (Table 1) are produced by the US rendering industry. Though the rendered products are used in many chemical and cosmetic industries, the major use of rendered products is in the animal feed industry, which produces products such as meat and bone meal (MBM). The MBM is an important by-product of rendering process and is considered to be an excellent source of nutritional elements such as protein, amino acid, calcium, and phosphorus. Although the use of MBM for feed has been debated (Sparks, 2002), the MBM has been used for many decades and fed to cattle without any restrictions. In certain conditions, the use of MBM as animal feed is screened because of the potential linkages between MBM and the spread of Bovine spongiform encephalopathy (BSE) (O’Brien, 2000; Food and Agriculture Organization (FAO), 2015a,b,c).

**TABLE 1:** Rendered product production in the US (data source: Market report by Swisher, 2014).

**Rendered products**	**2010**	**2011**	**2012**	**2013**	**2014**
	**1,000 metric tons**				
Inedible tallow	1, 511.20	1, 486.80	1, 453.20	1, 442.20	1, 356.70
Edible tallow	827.6	886.7	812	805.8	737.8
Lard	61.4	62.2	63.7	63.3	62.3
Yellow grease	868.8	906.4	884.4	900.8	868.8
Poultry fat	471.4	475.2	474.8	481.5	488.2
Poultry byproduct meal	1, 178.60	1, 188.10	1, 186.90	1, 203.80	1, 220.60
Feather meal	603.5	608.5	608	616.6	625.2
Meat and bone meal (MBM)	2, 244.70	2, 272.90	2, 261.50	2, 250.00	2, 116.00
Others	511.3	518.4	530.2	527.4	582.1
Total	8, 278.50	8, 405.20	8, 274.70	8, 291.40	8, 057.7

The MBM from cattle is, however, fed to non-ruminants and MBM from non-ruminant animals is fed to cattle in the US if MBM is free of cattle material prohibited in animal feed (CMPAF) (U.S. Food and Drug Administration (FDA), 2015a, b). The CMPAF includes the brain and spinal cords from cattle of 30 months of age and older. The entire carcass of cattle is not inspected and passed for human consumption unless the cattle are <30 months of age or the brains and spinal cords have been removed. In addition, the entire carcass of BSE-positive cattle, tallow derived from BSE-positive cattle, tallow derived from CMPAF that contain more than 0.15% insoluble impurities, and mechanically separated beef derived from CMPAF (U.S. Food and Drug Administration (FDA), 2008) are also included in CMPAF.

It is widely acknowledged that if infectious prions are present in animal feed through the use of rendered products from diseased animals as feed ingredients, then these prions are likely to enter animals through the feed. Subsequent consumption of meat of these animals will be likely to transmit prions to human. Though there is no direct evidence showing the primary routes of human exposure to prions via meat, the most likely route is through the consumption of the beef derived from prion infected cattle. The potential source of prion infections in these cattle is animal feed containing rendered animal proteins of infected diseased cattle (Sapkota et al., 2007).

The rendering industry lessens the burden on landfills, which may receive enormous number of animal mortalities and inedible portions of animal produced in animal industries if options of rendering are not available (Agriculture Workgroup (AgWG), 2018). As an example, during slaughtering and processing of animals (i.e., cattle, beef, sheep, pork, poultry, and fish), ∼37–49% of the live animal weight is removed that requires disposal or recycling. The production of inedible by-products from animal production industries in the US is about 54 billion pounds per year (Hamilton et al., 2012). If these by-products are not recycled or converted into edible form, all the remaining materials produced in animal industry and slaughter houses will likely to end up in landfills (Sparks, 2002; Hamilton et al., 2012). Currently, more than 20 billion pounds of valuable products are produced annually in the US through rendering process from normally-inedible animal products in the US (Hamilton et al., 2012). Considering the value of rendering industry in disposing off the animal carcasses and controlling consequential pollution and diseases risk caused by animal mortalities, the role of rendering industry is pivotal (Troutt et al., 2001; Hamilton et al., 2012; Suloma et al., 2012). Hence, improved understanding of the risks of infectious agents that may affect rendered products is important. The goal of this paper is to provide a comprehensive understanding of the risk of infectious agents of rendering industry, such as prions (infectious protein), *Salmonella enterica*, pathogenic *E. coli*, and *Listeria monocytogenes.*

## Risk of Infectious Agents in Rendered Products

The presence of infectious agents, such as prions, *Salmonella* spp., pathogenic *E. coli*, and *Listeria monocytogenes* can potentially compromise the public, and animal health. Though the elevated temperatures depending on the type of rendering processes used in rendering industry for recycling dead animals and slaughter waste has a potential to kill microbial pathogens, additional microbial safety measures may be needed for reducing the risk of microbial contamination. Timely transportation of dead animals from a farm to a rendering plant is often challenging. Steam heating, which is common in the US, uses temperature between 115°C and 146°C for 90 min to cook the raw animal tissues. Other rapid heating method could be infrared heating, which can be a great tool to provide a high heat transfer rate (Vidyarthi, 2017; Vidyarthi et al., 2019a). Despite the fact that the heat of rendering process is lethal to many infectious agents, the risk of contamination in rendered products cannot be ruled out. Infectious agents such as prion protein which are responsible for BSE, can survive in rendering process.

### Bovine Spongiform Encephalopathy (BSE)

Transmissible spongiform encephalopathies (TSEs) are a group of degenerative brain disorders, which affect mammals. The TSEs degenerate brain with severe neurological symptoms (U.S. Food and Drug Administration (FDA), 2015a, b; World Health Organization (WHO), 2015). The TSE affects a number of species, including ruminants (cattle, sheep, goats), cervides (deer, elk, moose), human, and felines. The human forms of the disease include Cresutzfeldt-Jakob disease (CJD) and its variants. The animal forms of the disease associated with TSEs include BSE in cattle, scrapie in sheep and goats and chronic wasting disease (CWD) mainly in deer, elk and moose (Stack et al., 2004). BSE is a fatal neurological disease in animals, and rendered products were found to be linked with BSE (Taylor et al., 1995; Taylor, 2000; Taylor and Woodgate, 2003). These diseases are associated with the accumulation of a protease-resistant, disease-associated isoform of the prion protein [called PrP (Sc)] in the central nervous system and other tissues (Greenlee and Greenlee, 2015). The prions are heat resistant and can survive rendering process (United States Department of Agriculture (USDA), 2013). The TSEs are relatively resistant to chemical and physical inactivation procedures (Taylor, 1989, 1993, 1999; Taylor et al., 1995; Prusiner, 1996; Chesebro, 1998; Horn, 2001; Taylor and Woodgate, 2003). BSE was recognized to be originated from England in the mid-1980s but later appeared in France, the Republic of Ireland, Portugal, and Switzerland. In the recent years, it has been detected to varying degrees in all the European Union (EU) countries except Sweden, and in some non-EU countries, such as Israel and Japan, mainly due to the import of BSE-infected products from EU (Taylor and Woodgate, 2003).

In 1986, when BSE was first discovered in the United Kingdom (UK), it was considered as a new disease, and the potential impacts on animal industries were not foreseen, and situation was exacerbated. Despite the multiple efforts of the UK government (beginning from 1988) to control the risk of BSE and damaging impacts on industry, the incidence of BSE peaked in 1992. In 1992, more than 36,700 cases were confirmed. A research in 1987 found a causal relationship between BSE and the use of MBM in ruminant feed (O’Brien, 2000). This triggered a widespread concern about MBM uses for animal feed. Subsequently, the use of the MBM for animal feed was banned in the UK. The implementation of the MBM restrictions were extended gradually to all farm animals in the UK. The old stocks of feedstuff manufactured before the ban were also withdrawn (O’Brien, 2000). As a result of BSE incidences, beginning from 1996, all farm animals including fish in the UK have been restricted from eating mammalian MBM. In addition to BSE, scrapie, which is another form of TSE, affects the central nervous system of sheep and goats, was linked to rendered products (BSE Info, 2015; Greenlee and Greenlee, 2015).

**FIGURE 2:**
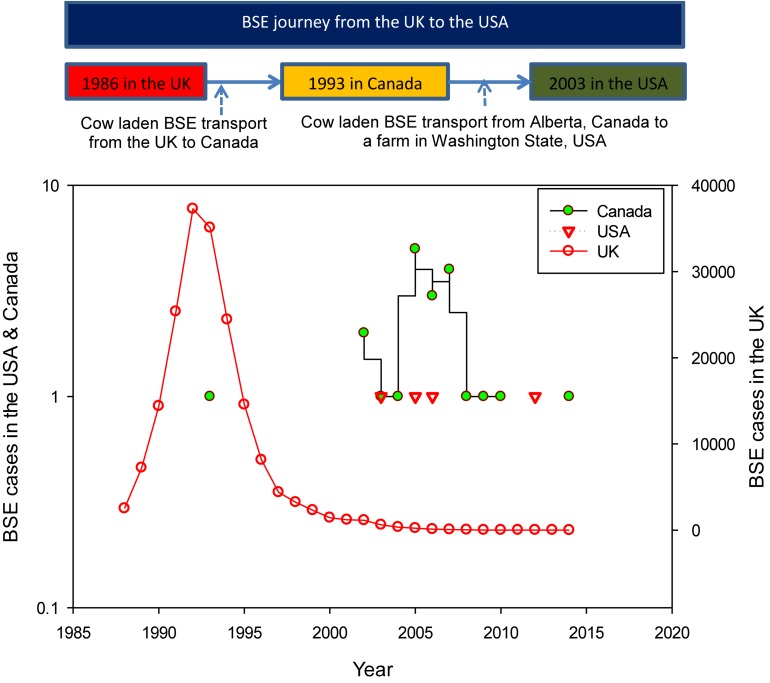
The BSE journey from the UK to the US via Canada (top), and the BSE cases in the UK, Canada, and the US (bottom) (data source of BSE cases: World Organization for Animal Health, 2015).

After BSE was first discovered in cattle in the UK, source tracking studies were conducted, and results indicated that BSE was transported from the UK to the US via Canada (Figure 2). The incidences of BSE in the UK, Canada, and the US are shown in Figure 2. Between 1986 and 2014, the total reported cases of BSE were 184,179, 21, and 4 in the UK, Canada, and US, respectively (World Organization for Animal Health, 2015). The implications of BSE were severe in the UK as well as in other countries including Canada and the US.

**FIGURE 3:**
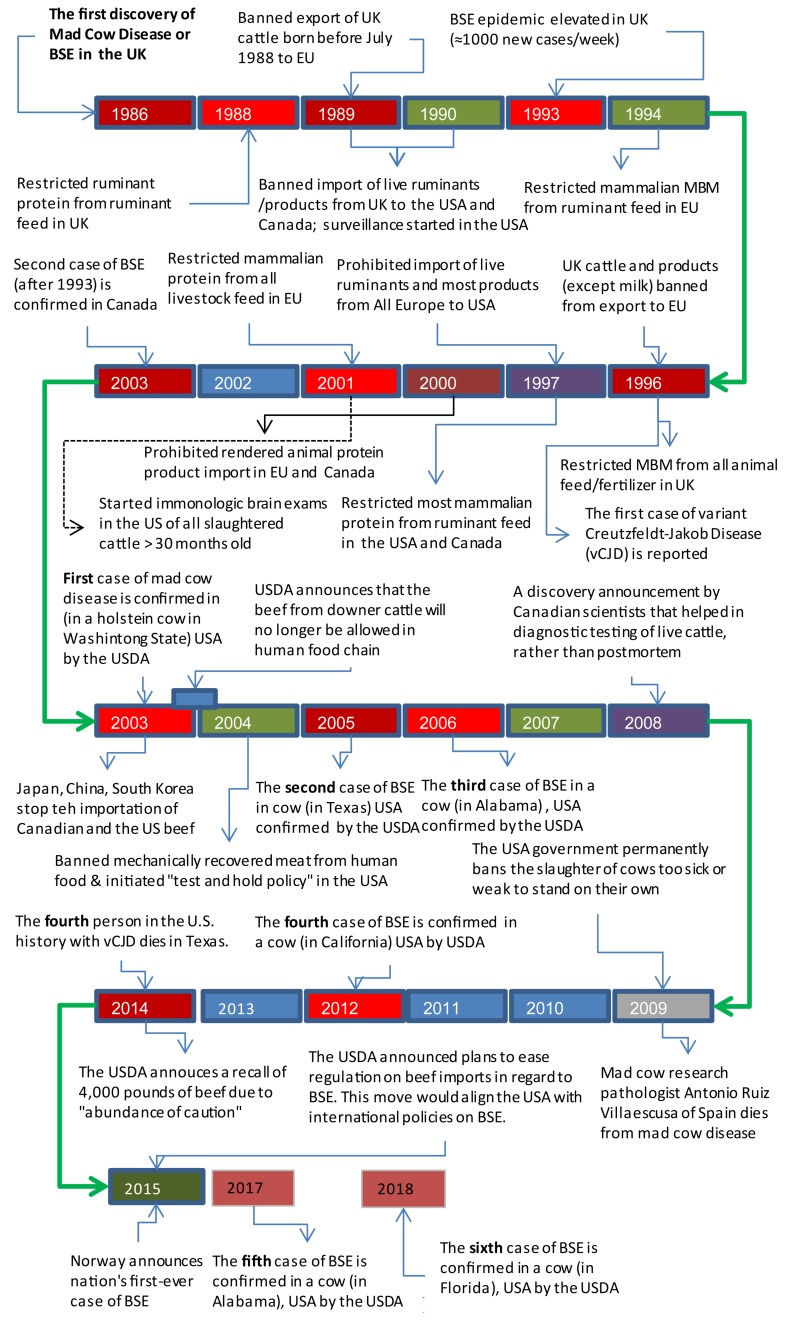
Time line and cases of BSE in the US, Canada, and the UK.

The disease timeline (1986–2015) describing the major BSE related events in the UK, Canada, and the US are shown in Figure 3. In 1993, the first case of BSE was diagnosed in Canada (Figure 3) in a cow (i.e., cow being raised for slaughter), which was imported from the UK in 1987 (Library of Parliament, 2005). Between 1982 and 1990, Canada imported 160 head of cattle from the UK, and 33% of those cattle (53 out of 160) were slaughtered and entered into the food chain. Out of those 160, the rendering plants received 16 cattle, and 11 cattle were exported to the US (Library of Parliament, 2005).

Further, in January 2003, a fed cow in Alberta was found lying down (incapable of rising), and symptoms of cow were qualified for BSE monitoring. The carcass was condemned and sent for rendering, where it entered into the animal food chain. In May 2003, the results of the fed cow’s head inspection showed that the animal had the BSE. During the same year (December 2003), the first case of BSE in the US was discovered in a farm in Washington State (Figure 3) and the DNA testing showed that the cattle was born in Alberta (Library of Parliament, 2005).

The incidences of BSE were alarming, and the economic losses caused by BSE were enormous. Animal industries in the UK, Canada, and the US suffered substantially due to BSE incidences. Initially, the losses in the UK were limited, however, when the potential link between the BSE and new variant Creutzfeldt-Jackob disease (CJD) was discovered, the beef and cattle industries in the UK were hit heavily (Atkinson, 1999).

In 1996, the domestic sales of beef products were declined by 40%, and export market was lost completely. Measures were taken to restore the public confidence in beef products and control the risk of BSE infected meat entering the food chain. One of the principal policy measures introduced in response to BSE was to support and improve rendering industry. The UK government paid renderers for the transport from abattoirs and the rendering of animals. During these years, rendering industry, which previously produced valuable rendered products, was transformed into waste disposal industry. Estimation showed that during the crisis (1996–1997), the total cost in additional public expenditure was ∼$1.5 billion, and the total economic loss to the UK from BSE crisis was $939–$990 million (Atkinson, 1999). A study by Atkinson (1999) estimated that the cumulative budgetary cost of BSE crisis in the UK was more than $3.8 billion.

Similar to the UK, economic losses caused by BSE were substantial in Canada. The impacts of the BSE on the beef industries of Canada and the US were significant. Many farm families in Canada lost total income by 33%, and revenue from international exports of live cattle calves was dropped by 67% (Library of Parliament, 2005). After the discovery of BSE in 2003 in one cow in Canada led to a decision by more than 40 countries to restrict import of live ruminant animals (cattle, sheep, goats, bison, elk, and deer), meat products, and animal by-products from Canada. According to the Canadian Health Coalition, total economic impact from BSE was $6.3 billion (Mitura and Pietro, 2005). The export restrictions in 2003, due to the risk of BSE, affected the US livestock economy considerably. According to the International Trade Commission Report, the restrictions posed due to BSE risk costed the US cattle industry billions, and many of the major importers barred the US beef after the confirmation of mad cow disease, which triggered almost $11 billion in revenue loss between 2004 and 2007 (Reuters, 2008).

In Canada, prior to 1997, there were no restrictions on use of meat meal or bone meal in animal feed. However, beginning from 1997, it has been forbidden to feed ruminants with mammalian meat meal or bone meal – except for meal made exclusively from pork or horse (Library of Parliament, 2005). The meals that contain fish or chicken are still allowed in the cattle feed chain. Animal meals are still allowed for the feeding poultry, pork, and domestic animals (i.e., dogs and cats). No additional BSE-related measures apply to rendered products. In 2007, a series of enhancements to the feed regulations were implemented to protect animal and human health as well as to strengthen 1997 ruminant feed ban. In commercial inedible rendering, the processing of zoo animals, companion animals, non-food animals used in research, other non-food animals (including wild animals), and food animals used in research that are not approved for release were restricted in Canada. Food animals, food animal products and by-products, food animals used in research, and food by-products were permitted to process in commercial inedible rendering plants (Library of Parliament, 2005). To mitigate the risk of BSE in the US, Food and Drug Administration (FDA) of the US proposed the rule of animal feed in 2004–2005 after the first BSE case in 2003. The regulation prohibited the use of certain cattle origin materials in the animal feed. These materials include: (1) the brains and spinal cords from cattle of 30 months of age and older; (2) the brains and spinal cords from cattle of any age not inspected and passed for human consumption; (3) the entire carcass of cattle not inspected and passed for human consumption (when the brains and spinal cords have not been removed); and (4) tallow that is derived from the prohibited material (U.S. Food and Drug Administration (FDA), 2008).

In 2007, the FDA proposed to prohibit the use of certain cattle materials in (or in the manufacture) the drugs, biologics, medical devices intended for use in human and human cells, tissues, cellular and tissue-based products, and in drugs intended for use in ruminant animals (US FDA, 2007). The FDA also proposed new record keeping requirements for medical products for humans and drugs for ruminants that are manufactured from material from cattle. Further, in 2013, the FDA announced a comment reopening on use of materials derived from cattle in human food and cosmetics. The FDA amended the rule indicating the small intestine of cattle, formerly prohibited cattle material, could be used in human food and cosmetics if the distal ileum was removed by a specified procedure or one that the establishment could demonstrate is equally effective in ensuring complete removal of the distal ileum (US FDA, 2013).

### Salmonella

*Salmonella* is considered as the major foodborne pathogens. According to the US Centers for Disease Control and Prevention (CDC), each year one million foodborne illnesses and 19,000 hospitalizations in the US are caused by *Salmonella* (non-typhoidal) (Centers for Disease Control and Prevention (CDC), 2010; Scallan et al., 2011). While rendered products may not be directly related to foodborne illnesses, the risk of rendered product borne *Salmonella* entering into the food chain through animal feed cannot be discarded. Many previous studies showed the presence of *Salmonella* in rendered products (Patterson, 1969; Smeltzer et al., 1980; Mead et al., 1999; Franco, 2006). In 2010, more than 1,800 people in 11 states became ill, and subsequent investigation showed the source of the outbreak to eggs supplied by two Iowa egg farms (Wright County Egg and Hillandale Farms).

In principle, the temperature used in the rendering industry for processing animal carcasses can eliminate majority of microbial pathogens and the studies showed that the finished products often harbor pathogens and other harmful microorganisms, such as *Salmonella*. Considering the fact that the temperatures (>100°C) used during the rendering process destroy these bacterial species, identifying the possible source of bacterial contamination in end products is important in order to ensure the safety of rendered products. One possible source of the contamination (i.e., presence of *Salmonella* in the end products) could be a result of cross-contamination within rendering facility (or from the environment), food processing equipment and the incoming raw material. Previous prevalence studies showed that the raw material entering the rendering facility contained a high load of pathogenic bacteria such as *Salmonella* (Kinley, 2009; Kinley et al., 2010). Moreover, studies have shown that *Salmonella* can persist on food processing equipment and be transferred into the product upon contact (Kinley, 2009). A study by Kinley (2009) evaluated 200 finished meals obtained from various rendering facilities across the US to determine the prevalence of *Salmonella* and *Enterococci*. While 83% of the samples were detected with *Enterococci* accounting for 54% of the total bacterial count, 13 *Salmonella* serotypes isolates were identified with 16 distinct pulse field gel electrophoresis (PFGE) patterns. Further, the thermal tolerance studies revealed that the *D*-values of these *Salmonella* isolates were 9.27–9.99, 2.07–2.28, and 0.35–0.40 min at 55, 60, and 65°C, respectively. Considering that rendering processes use temperature greater than 100°C, these findings indicated that the *Salmonella* contamination of the finished rendered meals was likely due to the cross-contaminations between the environment or the incoming raw materials and the finished products.

Feed mills use rendered products in addition to plant-based products to produce finished feed (Sapkota et al., 2007). Rendered products are produced by converting slaughter waste and dead animals, which are not suitable for human consumption, into animal feed products with the use of grinding, cooking, and pressing processes (NRA, 2015). Subsequently, protein blenders mix animal and plant-based protein ingredients to produce animal feeds (GAO, 2000). If the animal protein produced in rendering plants is contaminated (i.e., pathogens are present) it can potentially transmit contamination into animal feed.

*Salmonella* presence in animal protein produced in rendering plants as well in feed mills is not uncommon. A research by McChesney et al. (1995) found that 56% of 101 protein-based feed samples collected from 78 rendering plants, and 36% of 50 vegetable protein-based feed samples collected from 46 feed mills were positive for *Salmonella enterica* (Sapkota et al., 2007). Another study by Dargatz et al. (2005) found *Salmonella* spp. in 24% of 175 samples of mixed feed collected from a cattle feedlot in Colorado. Hofacre et al. (2001) detected *Salmonella* spp. in 14% of meat and bone meal samples, which were collected from a poultry feed mill.

Elevated levels of *Salmonella* in animal feed ingredients produced in rendered plants as well as in feed mills have a potential to contaminate animal feed. Developing improved surveillance and monitoring programs to quantify the levels of *Salmonella* entering human food chain through animal feed ingredients produced in either rendering plants or feed mills will likely to help in assessing the potential *Salmonella* risks to human and animals. Such attempts will likely to encourage rendering and animal feed industries for developing improved processes capable of controlling *Salmonella* levels in animal feed ingredients consumed by animals raised for human food. In addition, all the facilities producing animal feed need to be in compliance with the FDA Food Safety Modernization Act (FSMA, 2018). New guidance for the preventive controls for animal food for establishing and implementing improved supply-chain were introduced in 2017. FSMA and new rules for animal feed will likely to minimize the risk of *Salmonella* spread and reduce the risk of microbial contamination in animal food.

### Escherichia coli (E. coli)

*E. coli* is a type of gram-negative bacteria that normally lives in the gut and intestines of living beings, including humans and animals. Most *E. coli* strains are considered harmless; however, the *E. coli* O157:H7 is referred as one of the most pathogenic serotypes for humans. Infection of *E. coli* O157:H7 may cause serious and life-threatening complications in the lining of the gastrointestinal (GI) tract (CDC, 2019). A study by Saxena et al. (2015) reported that *E. coli* O157:H7 associated diseases and waterborne outbreaks resulted in high morbidity and mortality worldwide.

Currently substantial numbers of studies are available showing the potential presence of *E. coli*, including *E. coli* O157:H7 (Davis et al., 2003; Sargeant et al., 2004; Dargatz et al., 2005; Sapkota et al., 2007) in animal feeds. Although, this serotype is considered harmless for cattle because it does not bind to the walls of their GI track, the shedding of *E. coli* O157:H7 through the animal feces can contaminate the animals’ skin, hide, feathers, and their housing environment (Callaway et al., 2009). As an example, Sargent et al. (2001) isolated *E. coli* O157:H7 from 14.9% of 504 cattle feed samples. Dargatz et al. (2005) and Lynn et al. (1998) found *E. coli* in 48.2% of 1,070 cattle feed samples and 31.1% of 209 cattle feed samples, respectively. Further investigation by Dargatz et al. (2005) showed that many isolates were resistant to antibiotics i.e., 38.7, 24.7, 16.6, and 12.1% of 514 *E. coli* isolates were resistant to cephalothin, ampicillin, cefoxitin, and amoxicillin, respectively. A study by Hofacre et al. (2001) sampled 165 rendered animal protein products originating from poultry, cattle, and fish, and found that 85% of feed ingredient samples contained bacteria that were resistant to antibiotics such as amoxicillin, ampicillin, and clavulanic.

Other studies, such as Kinley (2009) and Laban et al. (2014) also assessed the *E. coli* (generic and pathogenic) contamination in rendered products. Kinley (2009) evaluated 150 finished rendered samples collected from several rendering plants across the US. The samples included feather meal, meat meal, MBM, poultry MBM, poultry meal, and blood meals. The study found *Salmonella* in 8.7% of the samples; however, no *E. coli* was detected. The study by Laban et al. (2014) collected 10 finished samples in Egypt from 10 poultry rendering operations where the samples were subjected to 2 bar pressure at 140°C for 40–90 min in dry batch cookers during the rendering process. The authors reported that the 10 and 20% of finished products were positive for *Salmonella* spp., and *E. coli*, respectively.

In general, *E. coli* is heat sensitive and elevated temperature (115–140°C) used in rendering process is likely to destroy *E. coli* levels if heat distribution is uniform in heating device in such a way that each particle is heated to rendering temperature. The detection of *E. coli*, in finished rendered products, however, suggests that additional safeguard measures are needed in order to eliminate *E. coli* completely in rendered products. Recently, an increased emphasis has been given to control antibiotic resistant *E. coli* entering food chain, which cannot be achieved without controlling antibiotic resistant *E. coli* levels in animal feed ingredients. Currently more than 110,000 and 60,000 illnesses are caused by *E. coli* non-O157 and *E. coli* O157, respectively, each year (Centres for disease control and prevention (CDC), 2011; Scallan et al., 2011). Any additional measures with the capability of safeguarding animal feed ingredients including rendered products from *E. coli* will likely to help in breaking the circulation path for *E. coli* in animal and human food chains and hence mitigate the public health risk.

### Listeria monocytogenes

In addition to *Salmonella* and *E. coli*, *Listeria monocytogenes (L. monocytogenes)* is one of the leading bacteria causing deaths from foodborne illnesses in the USA. *Listeria* is ubiquitous and persists in the environment for a long time, which may lead to contamination of animal feed, animals and rendering products. According to CDC report (2011), *L. monocytogenes* is responsible for 250 deaths and 1,600 illnesses in the USA each year. Approximately 16.6% of the 1,500 hospitalized people (due to *L. monocytogenes*) die each year (Centres for disease control and prevention (CDC), 2011). Due to high death rate of patients infected with *L. monocytogenes*, controlling *L. monocytogenes* in human and animal food chains is important to protect the public and animal health.

Currently, limited information describing the persistence of *L. monocytogenes* in rendering process exists. Previously, animal feed samples positive to *Listeria* spp. are reported. A study by Skovgaard and Morgen (1988) reported *L. monocytogenes* presence in 62% of 39 tested feed samples collected from seven different dairy farms. The authors also reported the mastitis caused by *Listeria* in cows. Ojeniyi et al. (1996) reported *L. monocytogenes* in poultry and poultry products. Further 28% of minced beef samples (collected from retail shops) were *L. monocytogenes* positive. Foegeding and Leasor (1990) studied heat resistance and growth of *L. monocytogenes* in liquid whole egg. The authors showed the similarity between *L. monocytogenes* inactivation in whole egg and raw milk and reported 2–3 log cycle reduction (99–99.9%) at 60°C in 3.5 min. The temperature used in rendering process is beyond the pasteurization temperature (60–70°C); however, previous studies showed *Listeria* spp. in rendered products.

Troutt et al. (2001) studied the raw and finished rendered products and reported 76.2 and 8.3% of raw products were positive to *Listeria* spp. and *L. monocytogenes*, respectively. The finished rendered product was not positive to *L. monocytogenes*; however, *Listeria* spp. was observed in 2.4% of the final rendered products. In another study, conducted by the Veterinary Laboratory Investigation and Response Network (Nemser et al., 2014), the authors found 66 samples (out of 480 samples including raw dog and cat goods, exotic animal feed, and jerky-type treats purchased online) positive to *Listeria* spp. and 48% of 66 samples were positive to *L. monocytogenes*. In 2011, a study in California, Singleton et al. (2012) evaluated raw horsemeat diets in zoo settings and found one out of 54 samples were positive for *Salmonella* (*Listeria* was not screened). Andreoletti et al. (2008) reported the risk of *Salmonella* spp. in animal feed is greater than the risks of *L. monocytogenes* and *E. coli* in animal feed. However, Nemser et al. (2014) suggested that the raw pet food products could be contaminated with either *Salmonella*, *L. monocytogenes*, or both.

## Potential Challenges in the Human and Animal Feed Safety

Tremendous data and information to ensure the safety of the food have been generated by various national and international regulatory and surveillance agencies including CDC, US Department of Agriculture (USDA), Animal and Plant Health Inspection Services (APHIS), Agriculture Research Service (ARS), Food Safety and Inspection Service (FSIS), FDA, Global *Salmonella* Survey by World Health Organization, Canadian Food Inspection Agency (CFIA), Food Standards Agency (FSA) in UK, State Food and Drug Administration of China (SFDA), Food Safety and Standards Authority of India (FSSAI), and Ministry of Health, Labor and Welfare (MHLW) in Japan. One of the potential challenges in implementing the existing food safety laws and monitoring the food quality is collecting the representative samples for quality testing out of sheer amount of food and animal proteins produced globally. In 2010, the world’s total cattle, sheep, and goats population was 11.8 billion. Poultry and pig population was 68.8 billion and 1.5 billion, respectively (Food and Agriculture Organization (FAO), 2015a,b,c; Statista, 2015). In 2014, the number of cattle slaughtered in the US, China, India, Canada, and Japan were 30.8, 49.2, 37, 3.2, and 1.1 million, respectively. The two countries (US and Brazil) alone produced more than 20 metric tons of beef and veal meat in 2014 (Index mundi 2015). According to the FAO (Food and Agriculture Organization (FAO), 2015a, b, c), bovine, poultry, and pig meat production in 2014 was 68, 108, and 115.5 million tons, respectively.

Considering the enormous livestock numbers, animal mortality, and number of slaughtered animals, large quantities of animal protein, feed, and waste is produced annually. With this sheer volume, implementing the existing food safety rules from farms to production sites to distribution centers is a key for controlling infectious agents in animal and human feed and maintaining high quality food in global food chain. The safety of human and animal food is likely to depend on how well existing laws are followed in various stages of food productions.

Increasing public health awareness, microbial safety of food supplies and associated cost has been a matter of global concern. It is a widely accepted fact that the safe food supplies reinforce sustainable development by not only supporting national economies, trade, and tourism but also food and nutrition security. Safety and security of food and nutrition for human and animal is important for a healthier generation. In spite of regulations in many countries related with food safety, issues such as monitoring, compliance, modern technology for contamination detection, safety certifications, consumer education and outreach on food safety, and the outbreaks of foodborne diseases are frequently heard (Kasbekar, 2018). These concerns are equally important to the end products obtained from animal rendering. The quality of feed ingredients produced by the rendering industry plays an important role in today’s complex food chain system. Therefore, it is expected that the rendering industries are cognizant of their responsibility for applying food safety programs at every level of production to ensure the absence of potentially hazardous contamination during the by-product production (Franco, 2006).

Supply of a safe in-feed ingredient to the rendering plant is still a massive challenge. Transportation of animal mortalities to the rendering facility in a timely fashion before the occurrence of decomposition is a major concern. Especially, during an outbreak of disease, transport and travel restrictions will be extremely challenging for renderers to obtain the in-feed material before starting the decay process. Further, animals killed as a result of a natural disaster, such as a hurricane, might not be accessible before they are excessively putrefied (USDA-APHIS, 2004). In those cases, achieving the overall food safety objectives related to rendered products becomes extremely vital.

Therefore, a proactive testing for pathogens and toxins prior to, during and post-rendering that influence the end product integrity is critical. Inclusion of good manufacturing practices (GMP), hazard analysis and critical control point (HACCP) and FMSA rules, promotion of the robust APPI Code of Practice, and carefully constructed third party food safety certification audit and long-term commitment and accountability could play a significant role in achieving sustainable food safety (Franco, 2006).

The outdated rendering plants and processing equipment such as cooker, pipelines, conveyer system, grinding and pressing system might pose a safety hazard to the workers and a challenge for achieving good quality end products. In past, some safety related catastrophic failures of processing vessels in the rendering plants were reported, including leaking of a jacketed steam-cooker, absence of safety valves and vessel explosion causing casualties (NBIB, 1987). Therefore, the existing plants need to be inspected on the regular basis thoroughly and updated with the modern facilities construction and technologies. The new or existing constructions must incorporate sanitary operations and environmental conditions, prevent the spread of contaminated and lethal microorganisms and malodorous condition, and provide sufficient space for the storage of carcasses, ancillary materials and finished products. Plant structures design should allow adequate cleaning, sanitation and maintenance, prevent adulteration of raw materials, and implement appropriate odor control systems (USDA-APHIS, 2004). All these will contribute to attain the safe production of the finished rendered products. In current animal-agriculture system, rendering industry is vital and it is linked to the whole food chain system. Implementing the aforementioned regulations alone will not be sufficient to restrain the complex food safety issues related to the rendered product. To eliminate the chances of microbial contamination through rendered products, food safety policies related to the rendered products must use a holistic approach which not only involves applying high temperature for rendering but also implementing protocols for regular monitoring and evaluation of rendered products (at multiple stages from production to transport) based on sound and proven science. It is important to connect all the possible links of the accompanying food chain system and define the roles and responsibilities of all the individual entities connected to pre- and post-rendering process.

## ConsEnt for Publication

All authors gave their consent for publication.

## AuthOr Contributions

PP formed the ideas for the manuscript and completed first draft. SV and AP critically reviewed and revised manuscript. VV, CV, BW, and MP provided assistance in review and correction. All authors read and approved the final manuscript.

## Conflict of Interest

SV was employed by The Morning Star Company.

The remaining authors declare that the research was conducted in the absence of any commercial or financial relationships that could be construed as a potential conflict of interest.
